# Impaired surface expression and conductance of the KCNQ4 channel lead to sensorineural hearing loss

**DOI:** 10.1111/jcmm.12080

**Published:** 2013-06-11

**Authors:** Yanhong Gao, Sergey Yechikov, Ana E Vázquez, Dongyang Chen, Liping Nie

**Affiliations:** a Department of Otolaryngology, University of California DavisDavis, CA, USA

**Keywords:** KCNQ4, hearing loss, mutations, potassium channel, surface expression, molecular chaperone, trafficking

## Abstract

KCNQ4, a voltage-gated potassium channel, plays an important role in maintaining cochlear ion homoeostasis and regulating hair cell membrane potential, both essential for normal auditory function. Mutations in the *KCNQ4* gene lead to DFNA2, a subtype of autosomal dominant non-syndromic deafness that is characterized by progressive sensorineural hearing loss across all frequencies. Despite recent advances in the identification of pathogenic *KCNQ4* mutations, the molecular aetiology of DFNA2 remains unknown. We report here that decreased cell surface expression and impaired conductance of the KCNQ4 channel are two mechanisms underlying hearing loss in DFNA2. In HEK293T cells, a dramatic decrease in cell surface expression was detected by immunofluorescent microscopy and confirmed by Western blot for the pathogenic KCNQ4 mutants L274H, W276S, L281S, G285C, G285S, G296S and G321S, while their overall cellular levels remained normal. In addition, none of these mutations affected tetrameric assembly of KCNQ4 channels. Consistent with these results, all mutants showed strong dominant-negative effects on the wild-type (WT) channel function. Most importantly, overexpression of HSP90β, a key component of the molecular chaperone network that controls the KCNQ4 biogenesis, significantly increased cell surface expression of the KCNQ4 mutants L281S, G296S and G321S. KCNQ4 surface expression was restored or considerably improved in HEK293T cells mimicking the heterozygous condition of these mutations in DFNA2 patients. Finally, our electrophysiological studies demonstrated that these mutations directly compromise the conductance of the KCNQ4 channel, since no significant change in KCNQ4 current was observed after KCNQ4 surface expression was restored or improved.

## Introduction

The KCNQ family of voltage-gated potassium channels (K_V_7) plays important roles in brain, heart, kidney and the inner ear. Mutations in four of the five KCNQ channels cause inherited human diseases, including cardiac arrhythmia, epilepsy, deafness, *etc*. 1–3. The expression of KCNQ4 (K_V_7.4) is predominantly detected in the inner ear and the central auditory pathways 4–7. In the inner ear, KCNQ4 is highly expressed in the basal membrane of sensory outer hair cells, where it mediates the M-like potassium current *I*_K,n_ 4–10. The activity of the KCNQ4 channel is crucial for the maintenance of hair cell membrane potential and for the K^+^ recycling in the cochlea 10–12 For the latter, KCNQ4 provides a major K^+^ efflux pathway from outer hair cells 11, 12. In the mouse model, loss of KCNQ4 function leads to degeneration of outer hair cells and progressive sensorineural hearing loss without vestibular phenotypes 13. KCNQ4 expression was also detected in the vestibular sensory epithelium and certain tracts and nuclei of the brainstem, however, the physiological significance of the KCNQ4 channel in these cells is currently unknown 4–6. In human, mutations in *KCNQ4* cause DFNA2, a subtype of autosomal dominant non-syndromic deafness that is characterized by progressive sensorineural hearing loss 7, 13, 14. At young ages, hearing loss in DFNA2 patients is moderate and predominantly affects high frequencies. The hearing loss progresses, usually in less than 10 years, to more than 60 dB with middle and low frequencies also involved 14, 15. By the age of 70, all affected individuals in DFNA2 families have severe to profound hearing loss across all frequencies 14, 16, 17. There are currently no therapeutic treatments to prevent progressive hearing loss in these patients. Development of such treatments has been hampered by the lack of understanding of the molecular aetiology of DFNA2.

Over the last two decades, various pathogenic *KCNQ4* mutations have been identified in DFNA2 patients (DFNA2 mutations) 7, 15, 18–31. Among them, the missense mutations L274H, W276S, L281S, G285C, G285S, G296S and G321S are loss-of-function mutations 7, 19, 29, 32, 33. Specifically, electrophysiological studies in *Xenopus laevis* oocytes and various cell lines have shown that these mutations lead to loss of KCNQ4 currents 7, 19, 29, 32. Yet, the molecular mechanisms by which these mutations lead to loss of KCNQ4 currents are not well understood. Using immunofluorescent and biochemical approaches, Mencia and colleagues demonstrated that the mutation G296S led to diminished cell surface expression of the mutant channel with a strong dominant-negative effect on WT KCNQ4 channels 29. Trafficking deficiency of G296S was further confirmed by a separate immunofluorescent study 32. In the latter, Kim *et al*. also found that five other DFNA2 mutants, L274H, W276S, L281S, G285C and G321S, are trafficking deficient, although no experiment was conducted to determine whether or not the trafficking phenotype was dominant 32. However, contrary to these findings, a recent biochemical study demonstrated that the DFNA2 mutations, W276S and G285C, had no significant effects on cell surface expression of the KCNQ4 channel 19 Therefore, it is not clear whether trafficking deficiency is a common mechanism for loss of KCNQ4 function in DFNA2 patients.

Like other voltage-gated potassium channel, KCNQ4 channels consist of six transmembrane domain, a pore-forming region, and two intracellular termini. Most of the DFNA2 mutations are clustered around the pore region of the KCNQ4 channel 19, 27, 31. Specifically, L274H, W276S and L281S are located within the pore helix; G285C and G285S are substitutions of the first glycine in the signature sequence of the K^+^-filter (GYG); G296S is located in a group of five highly conserved amino acids connecting the pore-loop and the transmembrane domain S6; G321S is at the junction of the S6 domain and the C-terminal of the KCNQ4 channel. Given the structural and functional significance of the amino acids affected, it is not surprising that these mutations lead to loss of KCNQ4 function. However, the finding that loss of KCNQ4 currents may be caused by decreased cell surface expression is striking and opens a series of important questions in the molecular aetiology of DFNA2. What are the underlying mechanisms for the loss of KCNQ4 currents in DFNA2? Is it because of decreased cell surface expression or impaired conductance of KCNQ4 channels or both? Is decreased surface expression a consequence of degradation or intracellular retention of DFNA2 mutants? Is it possible that DFNA2 mutants are intracellularly retained otherwise functional? Could surface expression of DFNA2 mutants be restored? Most importantly, could restoration of KCNQ4 surface expression rescue the function of these mutant channels? The answers to these questions are fundamental to our understanding of the molecular mechanisms underlying hearing loss in DFNA2 and will lay important ground work for a rational design of therapeutic treatments.

Generation of KCNQ4 channels on the cell surface requires proper folding, assembly and trafficking of KCNQ subunits. Despite the functional significance of the KCNQ4 channel, little is known about the molecular mechanisms that control these processes. We have recently demonstrated that the cellular level of the KCNQ4 channel is regulated by the HSP90 chaperone pathway 34. HSP90 is an evolutionally conserved molecular chaperone that plays a central role in the structural maturation and trafficking of numerous proteins involved in signal transduction, including protein kinases, steroid hormone receptors, transcriptional factors, endothelial nitric oxide synthase (eNOS) *etc*. 35. HSP90 is also required for the folding and trafficking of various membrane proteins, such as the cystic fibrosis transmembrane conductance regulator (CFTR), the ClC-2 chloride channel 36, the voltage-gated potassium channel hERG and the ATP-sensitive potassium channel (K_ATP_) 36–39. Most importantly, recent studies have shown that manipulating HSP90 function can be used to rescue folding and trafficking of various mutant proteins for the treatment of human diseases 40–44. Our previous study in HEK293T cells showed that HSP90α and HSP90β are key players in the molecular chaperone network regulating the cellular level of the KCNQ4 channel 34. While overexpression of HSP90β promotes KCNQ4 biogenesis, overexpression of HSP90α facilitates ubiquitin-dependent degradation of the channel. Moreover, overexpression of HSP90β dramatically increased the abundance of not only the WT but also the mutant KCNQ4 channels on the cell surface. Cell surface expression of the KCNQ4 channel in HEK293T cells mimicking heterozygous conditions of two DFNA2 mutations, L274H and W276S, could be restored by overexpression of HSP90β 34.

In this study, we investigated the effects of seven loss-of-function DFNA2 mutations, L274H, W276S, L281S, G285C, G285S, G296S and G321S, on the overall cellular level and on the cell surface expression of the KCNQ4 channel using both immunofluorescent and quantitative biochemical approaches. We tested whether these mutations affect subunit interaction and whether they have dominant-negative effects on the WT KCNQ4 function. We also explored the potential of HSP90 overexpression in rescuing cell surface expression of DFNA2 mutants L281S, G296S and G321S. Finally, we tested whether the function of DFNA2 mutations can be rescued by restoration of KCNQ4 surface expression in HEK293T cells mimicking the heterozygous KCNQ4 condition of DFNA2 patients.

## Material and methods

### Chemicals and reagents

All chemicals were from Sigma-Aldrich (St. Louis, MO, USA); media and reagents for cell culture were from Invitrogen (Grand Island, NY, USA), unless otherwise indicated.

### Expression constructs

*KCNQ4* (NM_004700) was cloned in pCMV6-XL5 vector and then tagged with a Myc or a modified HA epitope in the first extracellular loop of the KCNQ4 channel as described previously 29, 32. These tagged KCNQ4 channels (referred to as Myc-KCNQ4 or HA-KCNQ4) exhibited normal channel properties 29, 32. Constructs of the mutant KCNQ4 channels were generated from the tagged WT constructs using the QuikChange Lighting Site-Directed Mutagenesis Kit (Stratagene, Santa Clara, CA, USA) and verified by DNA sequencing. For immunofluorescent microscopy and electrophysiological recordings, the WT and the mutant KCNQ4 channels were subcloned into the pIRES2-DsRed2 vector. In addition, molecular chaperones, HSP90β (NM_007355) was cloned in pCMV6-XL5.

### Antibodies

Primary antibodies used in this study were anti-HA (MMS-101P; Covance, Emeryville, CA, USA), anti-Myc (11667149001; Roche, Mannheim, Germany), anti-GAPDH (AM4300; Ambion, Austin, TX, USA), anti-HSP90β (sc-1057; Santa Cruz Biotechnology Inc., Santa Cruz, CA, USA). The secondary antibodies, including the anti-goat-horseradish peroxidase (HRP; 705-035-003), antimouse-HRP (715-035-151) and antimouse IgG-FITC (115-095-146) were from Jackson ImmunoResearch Laboratories Inc. (West Grove, PA, USA).

### Cell culture and transfection

HEK293T cells (Sigma-Aldrich) were used for all experiments. These cells were maintained according to the manufacturers' instruction. All transfection were carried out using Lipofectamine 2000 as described by the manufacturer (Invitrogen). Following transfection, the cells were incubated at 37°C for 24 hrs.

### Immunofluorescent microscopy

HEK293T cells were cultured on glass cover slips and transfected with KCNQ4 channels in pIRES2-DsRed2 (0.4 μg per well in 6-well plates). Twenty-four hours after transfection, the cells were fixed in 4% paraformaldehyde for 5 min., followed by three washes in PBS, and blocked in StartingBlock blocking buffer (Fisher Scientific, Pittsburgh, PA, USA) for 10 min. For permeabilization, the blocking buffer was supplemented with 0.1% Triton X-100. The cells were then treated with mouse monoclonal anti-HA antibody (1:500 dilution) for 1 hr and washed three times with PBS before incubation with goat-antimouse IgG-FITC (1:600 dilution, 1 hr). After a brief wash with PBST (PBS plus 0.05% Tween 20), the cells were incubated in the dark with DAPI for 5 min., followed by three washes with PBST, and then mounted in ProLong Gold Antifade Reagent (Invitrogen) on glass slides. Fluorescent images were captured using Carl Zeiss LSM510 confocal microscope. All procedures were performed at room temperature.

### Co-immunoprecipitation

Transfected cells were lysed in NP40 lysis buffer supplemented with protease inhibitor cocktail (P8340, Sigma-Aldrich), on ice for 30 min. Cell lysates were then cleared by centrifugation at 18,400 × *g* for 10 min. at 4°C and incubated with primary antibodies as indicated at 4°C for 16 hrs. The protein complexes were isolated and purified using Dynabeads Protein G (100-04D; Invitrogen) following manufacturer's protocol and analysed by Western blot (see below).

### Isolation of surface KCNQ4 proteins

Twenty-four hours after transfection, cells were washed twice with PBS *in situ* in 6-well culture plates and treated with mouse monoclonal anti-HA or anti-Myc antibodies (1:500 dilution) for 30 min. to label KCNQ4 channels on the cell surface. Following three washes with PBS, the cells were lysed with NP40 lysis buffer supplemented with protease inhibitor cocktail (P8340; Sigma-Aldrich), on ice for 30 min. The cell lysate was transferred to fresh tubes and centrifuged at 14,000 rpm for 10 min. at 4°C. Surface KCNQ4 proteins were captured using Dynabeads Protein G as instructed by the manufacturer. Proteins eluted from Dynabeads were analysed by Western blot (see below).

### Western blot

Proteins were separated on Criterion™ TGX Precast Gels (Bio-Rad Life Science, Hercules, CA, USA) and transferred to a nitrocellulose membrane (Bio-Rad Life Science). The membrane was probed with appropriate primary antibodies followed by incubation with HRP-conjugated secondary antibodies and visualized by SuperSignal West Pico Chemiluminescent Substrate (34077; Fisher Scientific). Chemiluminescent signals were collected by ChemiDoc XRP Imaging System and analysed by Quantity One software (Bio-Rad Life Science). Each band was quantified as the total pixel value after subtraction of the background and normalized to the loading control protein GAPDH.

### Whole-cell voltage clamp recording

HEK293T cells were trypsinized 24 hrs after transfection, seeded onto poly-L-lysine-coated glass coverslips, and maintained under normal growth condition for about 4 hrs. Before recording, cells were extensively washed with external solution (10 mM NaCl, 4.5 mM KCl, 2 mM CaCl_2_, 1 mM MgCl_2_, 10 mM HEPES, pH7.4, and osmolarity of 303 mmol/kg). Only healthy looking attached cells expressing DsRed fluorescent marker were used for recordings. Glass electrodes with resistance ranging from 1.5 to 3.0 MΩ and filled with internal solution (2.5 mM Na_2_ATP, 135 mM KCl, 3.5 mM MgCl_2_, 5 mM EGTA, 2.41 mM CaCl_2_, 5 mM HEPES pH7.2, and osmolarity of 300 mmol/kg) were used. Data acquisition was performed using a HEKA EPC-10 amplifier and HEKA PatchMaster software (HEKA, Bellmore, NY, USA) with high band Bessel filter set to 10 kHz and low band filter set to 0.2 kHz. The protocol consisted of holding for 1 sec. at −80 mV, followed by depolarization from −80 to +50 mV in 10 mV steps for 1.5 sec., −50 mV for 1 sec. followed by 1 sec. at −80 mV, with 5 sec. in between each testing sequence. Whole-cell current densities (pA/pF) were calculated as the maximal current (pA) divided by the cell capacitance, C-slow (pF). Each day at least five cells expressing WT KCNQ4 were recorded as internal control group. All recordings were performed at room temperature.

### Statistics

Biochemical results were presented as mean ± SD of at least three independent experiments. The measurements were statistically analysed using two-tailed unpaired Student's *t*-test (MS Excel 2010). Significance was reported as *P ≤ 0.05 and **P ≤ 0.01. Whole-cell current data were exported from PatchMaster and current traces were plotted using Origin v8.6 software (OriginLab Corp, Northampton, MA, USA).

## Results

### Effects of DFNA2 mutations on KCNQ4 expression

**Fig. 1:**
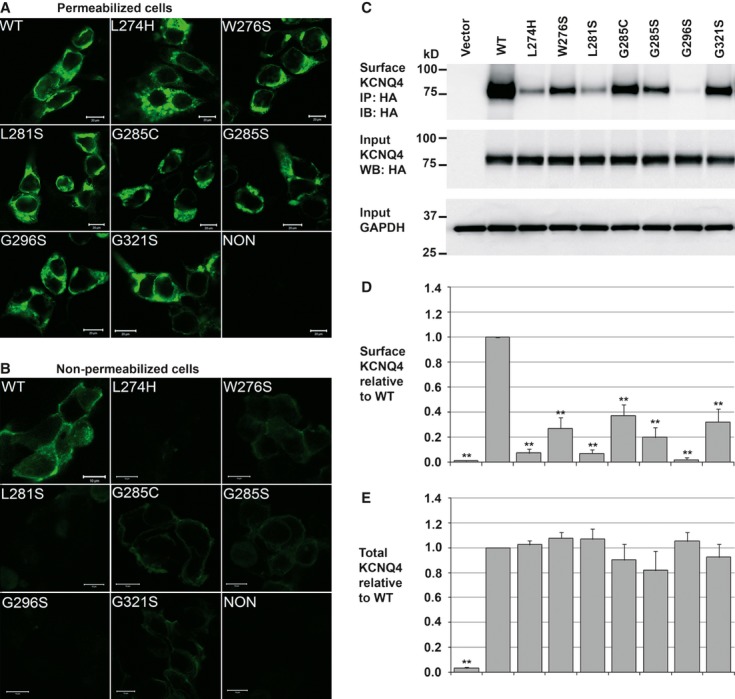
Effects of DFNA2 mutations on KCNQ4 expression. (**A** and **B**) Immunofluorescent microscopy. HEK293T cells were transfected with either the WT or a mutant HA-KCNQ4 (0.4 μg per well in 6-well culture dishes). After fixation, cells were blocked with or without permeabilization and treated with a mouse monoclonal antibody against the HA tag in the first extracellular loop of KCNQ4 channels, followed by incubation with a FITC-conjugated goat-antimouse secondary antibody. Cellular distribution of KCNQ4 channels (green) was determined by confocal microscopy. (**A**) In permeabilized cells, green fluorescent signal corresponding to total KCNQ4 expression was detected along the secretory pathway in all cells. (**B**) In non-permeabilized cells, green fluorescent signal corresponding to cell surface KCNQ4 channels was detected in cells transfected with the WT KCNQ4 channel, but diminished in those transfected with the mutant channels. Scale bars correspond to 20 μm in **A** and 10 μm in **B**. (**C**) Representative Western blot. Total and surface KCNQ4 channels were determined in transiently transfected HEK293T cells (0.4 μg per well in 6-well culture dishes). While cell surface expression of the mutant channels was significantly reduced (upper panel); the total amount of these channels remained unaffected (middle panel). (**D** and **E**) Summary data. Relative surface and total KCNQ4 expression normalized to the WT conditions, each data point in the bar graphs represents the mean ± SD of three experiments (*P ≤ 0.05, **P ≤ 0.01).

Seven loss-of-function missense mutations, L274H, W276S, L281S, G285C, G285S, G296S and G321S were generated in the human *KCNQ4* gene. Both the WT and mutant KCNQ4 channels were tagged with a HA epitope at the extracellular loop between the S1 and S2 domains and expressed in HEK293T cells. Immunofluorescent analyses were carried out using a primary antibody specific to the HA tag and a FITC-conjugated secondary antibody in both permeabilized and non-permeabilized cells. Confocal microscopy demonstrated strong green fluorescence with similar cellular distribution along the secretory pathway in all permeabilized cells expressing the WT or a mutant channel (Fig. 1A), suggesting that none of these mutations had an effect on overall KCNQ4 expression. However, in non-permeabilized cells, striking differences were observed between the WT and the mutant groups. Green fluorescent signal corresponding to cell surface KCNQ4 was robust in cells expressing the WT channel, but was significantly weaker in those expressing KCNQ4 mutants (Fig. 1B), indicating profound effects of these mutations on KCNQ4 surface expression.

To confirm these results, we further conducted quantitative biochemical analysis. Surface KCNQ4 channels were affinity-purified from HEK293T cells expressing either the WT HA-KCNQ4 or a mutant HA-KCNQ4 and analysed by Western blot. Compared to WT control, the relative amount of KCNQ4 mutants on the cell surface was significantly reduced, respectively, to 7.55 ± 2.32% (L274H), 26.48 ± 6.03% (W276S), 7.16 ± 1.93% (L281S), 35.77 ± 6.08% (G285C), 18.47 ± 5.30% (G285S), 1.78 ± 1.11% (G296S) and 31.51 ± 5.72% (G321S) (Fig. 1C and D). In contrast, no significant changes were detected in total KCNQ4 protein levels (Fig. 1C and E). These data indicated that all seven DFNA2 mutations tested disrupt the trafficking of the KCNQ4 channel to the cell surface, but not its overall cellular level.

### Effects of DFNA2 mutations on KCNQ4 subunit interaction

**Fig. 2:**
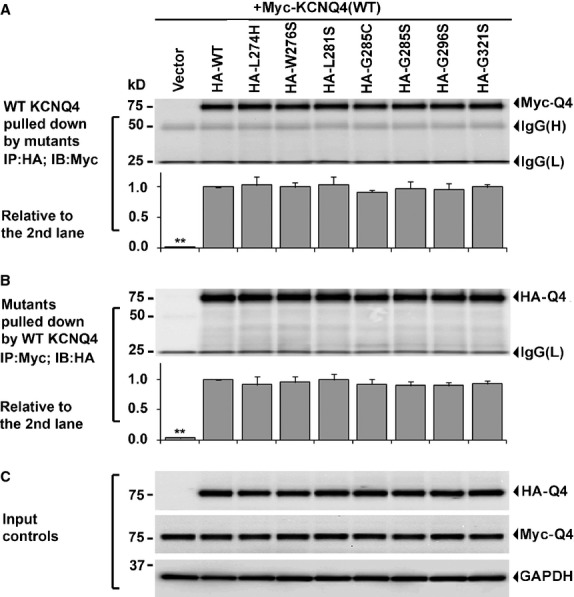
Effects of DFNA2 mutations on KCNQ4 subunit interaction Each HA-tagged mutant KCNQ4 channel was co-expressed with the Myc-tagged WT KCNQ4 in HEK293T cells at a ratio of 1:1 (0.2 μg each). Vector and the WT HA-KCNQ4 were also co-expressed with the WT Myc-KCNQ4 channel as positive and negative controls. The cell lysates were collected and used in reciprocal co-immunoprecipitation assay 24 hrs following the transfections. (**A**) The forward experiment – the WT Myc-KCNQ4 subunits co-precipitated with each mutant HA-KCNQ4 were analysed in parallel with the control groups. (**B**) The reverse experiment – the mutant HA-KCNQ4 subunits co-precipitated with the WT Myc-KCNQ4 were evaluated in parallel with the control groups. (**C**) The input – 5 μg of total protein was used in Western blot to test the amount of the WT Myc-KCNQ4, the WT HA-KCNQ4 and mutant HA-KCNQ4 subunits as well as GAPDH in each cell lysate. In summary, no significant difference between the WT and the mutant groups was detected in either forward or reverse experiment. Each data point in the bar graphs represents the mean ± SD of three experiments (*P ≤ 0.05, **P ≤ 0.01).

DFNA2 patients are heterozygous for the *KCNQ4* alleles, encoding equal amount of the WT and mutant KCNQ4 subunits. To examine whether DFNA2 mutations affect heteromeric assembly between the WT and mutant subunits, mutant HA-KCNQ4 channels were co-expressed individually with the WT Myc-KCNQ4 in HEK293T cells at a ratio of 1:1 to mimic the heterozygous condition of DFNA2 patients. The cell lysates were analysed by reciprocal co-immunoprecipitation assays. In forward experiments, interactions between the WT and mutant subunits were assessed by the amount of the WT Myc-KCNQ4 subunit co-precipitated with mutant HA-KCNQ4 subunits (Fig. 2A). In reverse experiments, the interactions were evaluated by the amount of mutant HA-KCNQ4 subunits detected in the Myc-WT KCNQ4 immunoprecipitates (Fig. 2B). Compared with the WT control (Fig. 2, second lanes), all mutant subunits showed normal abilities to interact with WT subunits, suggesting that tetramerization between the WT and these mutant subunits was not disrupted.

### Dominant-negative effects of DFNA2 mutations on WT KCNQ4 function

**Fig. 3:**
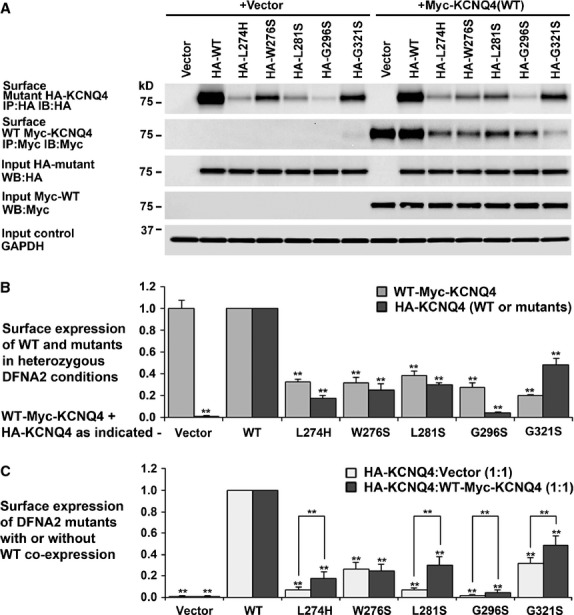
Surface expressions of the WT and mutant KCNQ4 subunits in cells mimicking the heterozygous condition of DFNA2 patients Each HA-tagged mutant channel was co-expressed with the Myc-tagged WT KCNQ4 channel in HEK293T cells at ratio 1:1 (0.2 μg each). The HA-tagged WT-KCNQ4 and vector were also co-expressed with the WT myc-KCNQ4 channel as controls. Surface KCNQ4 were affinity-purified and assessed by Western blot. (**A**) Representative Western blot. Surface expression of all mutant HA-KCNQ4 was improved, although to various degrees, by co-expression of the WT Myc-KCNQ4 channel (Top panel, IP:HA, IB:HA). In contrast, surface expression of the WT Myc-KCNQ4 channel was dramatically decreased in all cases (IP:Myc, IB:Myc), showing that not only the WT KCNQ4 cannot rescue cell surface expression of DFNA2 mutants in these cells but also mutations have dominant-negative effects on the WT KCNQ4 channel. (**B**) Summary data. Surface expression of the WT and mutant KCNQ4 subunits in cells mimicking the heterozygous condition of DFNA2 patients, relative to the WT value (WT HA-KCNQ4 plus WT myc-KCNQ4 at ratio of 1:1). (**C**) Summary data. Surface expression of mutant subunits (HA-tagged) with or without co-expression of the WT KCNQ4 subunit (Myc-tagged), relative to the WT value (see **B**). Each data point in the bar graphs represents the mean ± SD of three experiments (*P ≤ 0.05, **P ≤ 0.01).

**Fig. 4:**
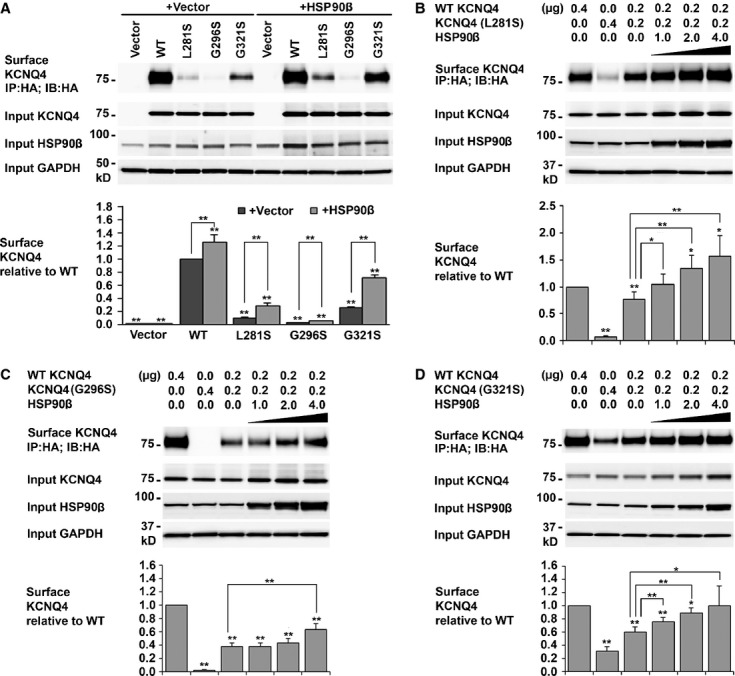
Rescue of KCNQ4 surface expressions by HSP90β. (**A**) KCNQ4 surface expression in cells co-expressing DFNA2 mutants and HSP90β. HEK293T cells were transfected with the indicated combination of plasmid DNAs (0.4 μg for each KCNQ4 channel and 1 μg for HSP90) and cultured for 24 hrs. Surface KCNQ4 channels were labelled with antibodies against the HA tag prior to cell lysis, affinity-purified using Dynabeads Protein G, and assessed by Western blot. Overexpression of HSP90β increased cell surface expression of KCNQ4 mutations by two- to fourfold. Each data point in the bar graphs represents the mean ± SD of three experiments (*P ≤ 0.05, **P ≤ 0.01). (**B**–**D**) Restoration of KCNQ4 surface expression in cells mimicking the heterozygous condition of DFNA2 patients by HSP90β. HEK293T cells were transfected with WT and a mutant KCNQ4 channel at ratio 1:1 plus various amount of HSP90β as indicated. Twenty-four hours after transfection, surface KCNQ4 channels were isolated and analysed as indicated in **A**. KCNQ4 surface expression was restored to the WT level in cells expressing WT/L281S/HSP90β (**B**), or WT/G321S/HSP90β (**D**); it was also improved considerably in cells expressing WT/G296S/HSP90β (**C**). Each data point in the bar graphs represents the mean ± SD of three experiments normalized to the WT value (*P ≤ 0.05, **P ≤ 0.01). Other significantly different pairs were indicated by lines.

Since DFNA2 mutants retained their ability to form heteromeric channels with the WT subunit and their overall expression levels remained unaffected, it was important to determine whether trafficking deficiencies of these mutants have dominant-negative effects on the WT subunit. We assessed the abundance of the WT KCNQ4 subunit on the surface of HEK293T cells mimicking the heterozygous condition of DFNA2 patients. Five DFNA2 mutants, L274H, W276S, L281S, G296S and G321S, were co-expressed individually with the WT KCNQ4 at a ratio of 1:1 and all of these mutants showed strong dominant-negative effects on the trafficking of the WT subunit, which was reduced, respectively, to 32.22 ± 3.04%, 32.12 ± 4.30%, 38.64 ± 4.41%, 27.65 ± 4.18% and 19.89 ± 1.46% of the normal level (Fig. 3A and B). Moreover, trafficking deficiencies of the mutant subunits could not be rescued by co-expression with the WT KCNQ4 subunits in these cells (Fig. 3A and B), even though the abundance of the mutant subunits on the cell surface increased significantly in four of the five cases (Fig. 3A and C). The total surface KCNQ4 in these cells, including homomeric WT tetramers, homomeric mutant tetramers and heteromeric tetramers containing both the WT and mutant subunits, were decreased to 39.6 ± 4.0% (WT/L274H), 60.9 ± 5.1% (WT/W276S), 76.3 ± 8.3% (WT/L281S), 37.7 ± 5.3% (WT/G296S) and 60.4 ± 8.2% (WT/G321S) of the normal WT level (Fig. 4B–D and 34). These *in vitro* data suggested that KCNQ4 surface expression in the sensory hair cells and neurons of heterozygous DFNA2 patients might be significantly lower than the normal level.

### Rescue of KCNQ4 surface expression by HSP90

Various approaches have been developed to rescue trafficking deficiency of pathogenic mutant channels. We have recently found that overexpression of the molecular chaperone HSP90β significantly improved cell surface expression of the WT KCNQ4 and mutant channels L274H and W276S 34. In this study, we first tested the effects of HSP90β overexpression on cell surface expression of homomeric mutant channels L281S, G296S and G321S in transfected HEK293T cells. Western blot showed that surface expression of the mutant channels significantly increased from 9.71 ± 1.75% to 28.91 ± 3.97% (L281S), 2.56 ± 0.27% to 5.44 ± 0.36% (G296S), 26.02 ± 1.17% to 52.01 ± 3.55% (G321S) respectively (Fig. 4A). Then, we tested whether cell surface expression of the KCNQ4 channel could be restored by HSP90β overexpression in HEK293T cells mimicking the heterozygous condition of DFNA2 patients. In this case, each mutant channel was co-expressed with the WT KCNQ4 at a ratio of 1:1 plus various amount of HSP90β. Our data showed that KCNQ4 surface expression in cells expressing WT/L281S or WT/G321S could be restored to the level comparable to that of the WT; in the cells expressing WT/G296S, significant improvements were also observed (Fig. 4B–D).

### Effects of DFNA2 mutations on KCNQ4 conductance

**Fig. 5:**
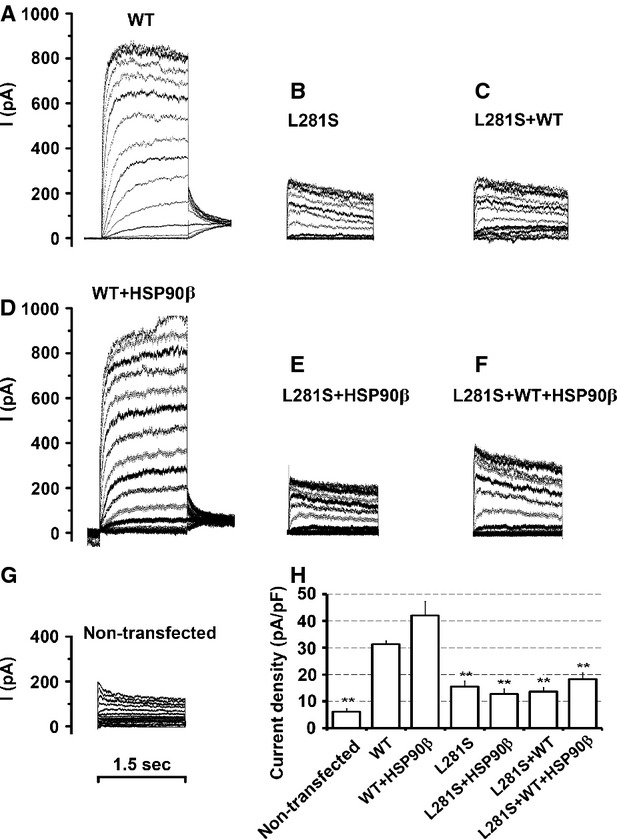
Functional rescue of the mutation L281S by HSP90β (**A**–**G**). Whole-cell current traces recorded, in response to depolarization from −80 to +50 mV in 10 mV steps in HEK293T cells transfected with the indicated combination of the KCNQ4 channel and HSP90β. (**A**) 0.5 μg WT KCNQ4+2.5 μg vector; (**B**) 0.5 μg L281S + 2.5 μg vector; (**C**) 0.25 μg L281S + 0.25 μg WT KCNQ4+2.5 μg vector; (**D**)0.5 μg WT KCNQ4 + 2.5 μg HSP90β, (**E**) 0.5 μg L281S + 2.5 μg HSP90β, (**F**) 0.25 μg L281S + 0.25 μg WT KCNQ4+2.5 μg HSP90β, (**G**) non-transfected cells as negative control. (**H**) Average current densities (mean ± SEM of 5–7 cells, *P ≤ 0.05, **P ≤ 0.01). The average current density of the mutant L281S was significantly lower than that of the WT channel. No significant differences in current densities were found in cells expressing L281S alone or co-expressing L281S/WT, L281S/HSP90β, or L281S/WT/HSP90β.

**Fig. 6:**
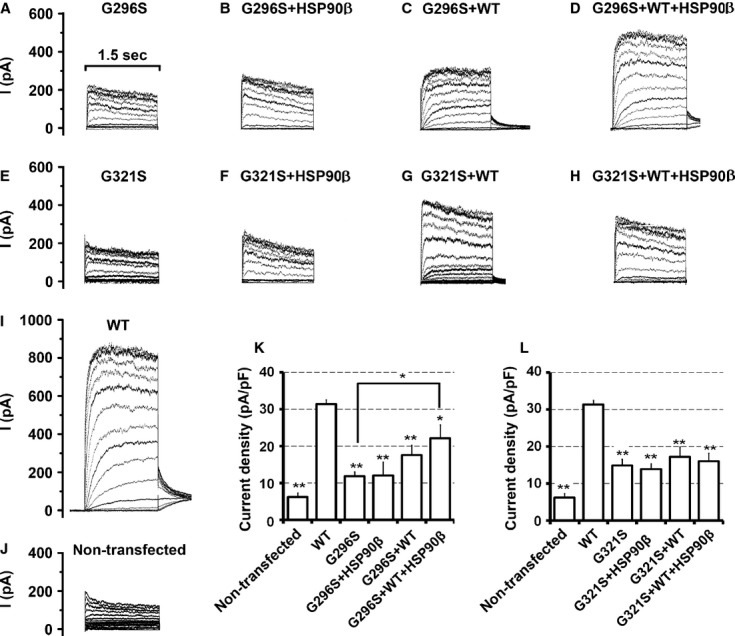
Functional rescue of the mutations G296S and G321S by HSP90β (**A**–**J**). Whole-cell current traces recorded in HEK293T cells transfected with the indicated combination of KCNQ4 channels and HSP90β, in response to depolarization from −80 to +50 mV in 10 mV steps. (**A**) 0.5 μg WT KCNQ4 + 2.5 μg vector; (**B**) 0.5 μg G296S + 2.5 μg vector; (**C**) 0.5 μg G321S + 2.5 μg vector; (**D**) 0.5 μg G296S + 2.5 μg HSP90β; (**E**) 0.25 μg G296S + 0.25 μg WT KCNQ4 + 2.5 μg vector; (**F**) 0.25 μg G296S + 0.25 μg WT KCNQ4 + 2.5 μg HSP90β; (**G**) 0.5 μg G321S + 2.5 μg HSP90β; (**H**) 0.25 μg G321S + 0.25 μg WT KCNQ4 + 2.5 μg vector; (**I**) 0.25 μg G321S + 0.25 μg WT KCNQ4 + 2.5 μg HSP90β; (**J**) non-transfected cells. (**K** and **L**) Average current densities (mean ± SEM of 5–7 cells, *P ≤ 0.05, **P ≤ 0.01), other significantly different pairs are connected with lines. Co-expression with WT or HSP90 or with WT plus HSP90 had no effects on the current densities of G296S or G321S. The average current density in the cells expressing G296S/WT/HSP90β is significantly higher than in cells expressing G296S alone but still lower than the WT channel level.

Whole-cell currents were recorded in transfected HEK293T cells to evaluate whether improved cell surface expression was sufficient for functional rescue of DFNA2 mutants. For each DFNA2 mutant, we conducted four sets of electrophysiological recordings from cells transfected with, (*i*) a DFNA2 mutant alone; (*ii*) a DFNA2 mutant plus HSP90β; (*iii*) a mutant and the WT KCNQ4 at a ratio of 1 to 1 to mimic the heterozygous condition of the DFNA2 patients; (*iv*) a mutant and the WT KCNQ4 at ratio of 1:1 plus HSP90β. Whole-cell currents were also recorded from cells expressing the WT KCNQ4 under the same conditions as control groups. Outward currents recorded from cells expressing the WT KCNQ4 alone were similar to whole-cell currents reported previously 7, 19, 29, 32, 45_._ Compared with these currents (Fig. 5A), whole-cell currents recorded from cells transfected with the DFNA2 mutant L281S, G296S or G321S were much smaller and similar to the background levels (Figs 5B, 6A and E). Specifically, the average current density was 31.37 ± 1.20 pA/pF (*n* = 30) for the WT channel, but 15.51 ± 2.01 pA/pF (*n* = 15) for the mutant L281S, 11.82 ± 2.7 pA/pF (*n* = 15) for G296S, 14.89 ± 1.77 pA/pF (*n* = 12) for G321S, and 6.31 ± 1. 01 pA/pF (*n* = 5) for non-transfected cells (Figs 5H, 6K and L). In cells mimicking the heterozygous condition of the DFNA2 patients, the average current densities were 18.26 ± 2.40 pA/pF (*n* = 11) for WT/L281S, 17.58 ± 2.7 pA/pF (*n* = 12) for WT/G296S and 17.28 ± 2.64 pA/pF (*n* = 7) for WT/G321S (Figs 5H, 6K and L), significantly smaller than the WT level (31.37 ± 1.20 pA/pF, *n* = 30). Our data demonstrated that the function of DFNA2 mutants could not rescue by co-expression of the WT KCNQ4 subunit; instead, the mutants had dominant-negative effects on the WT KCNQ4 currents (Figs 5B and C, 6A, C, E, and G).

Overexpression of HSP90β increased cell surface expression of the WT KCNQ4 channel by 26 ± 10.93% (Fig. 4A). In agreement with this result, the average current density in cells co-expressing the WT KCNQ4 and HSP90β was higher (42.07 ± 5.13 pA/pF, *n* = 6 compared to 31.37 ± 1.20 pA/pF, *n* = 30 for WT alone), although the difference did not achieve statistical significance (Fig. 5A, D and H). However, overexpression of HSP90β had no obvious effect on the whole-cell current of the DFNA2 mutant L281S, G296S, or G321S (Figs 5B, E and H, 6A, B, E, F, K, and L). Similarly, overexpression of HSP90β did not lead to higher current densities in cells mimicking the heterozygous condition of these mutants (Figs 5 and 6); despite significant improvements in KCNQ4 surface expression in these cells (Fig. 4). Notably, the average current density in cells expressing WT/G296S/HSP90β was significantly higher than in cells expressing G296S alone (22.15 ± 3.63 pA/pF, *n* = 8, compared with 11.82 ± 1.2 pA/pF, *n* = 12), but it is still much smaller than the WT level (Fig. 6K). Little or no increase in the average current densities after restoration of KCNQ4 surface expression indicates that the DFNA2 mutations L281S, G296S or G321S disrupt the conductance of the KCNQ4 channel.

## Discussion

To become functionally active, KCNQ4 subunits must fold and assemble to tetrameric channels in the endoplasmic reticulum (ER) and translocate to the plasma membrane. In human cells, these processes are controlled by a sophisticated molecular network and monitored by the protein quality control system 46–48. According to the current view of the ER quality control, only the proteins that have attained their native structures in the ER are exported efficiently into later compartments of the secretory pathway and transported to the plasma membrane; misfolded or misassembled proteins are retained in the ER, then dislocated across the ER membrane and degraded through ubiquitination-proteasome pathway, a process known as the ER-associated degradation 49, 50. Mutations in KCNQ4 channels may cause structural changes in KCNQ4 channels that affect their abilities to pass the ER quality control. However, our data demonstrated that none of the missense mutations tested, including L274H, W276S, L281S, G285C, G285S, G296S and G321S, affect the cellular level of the KCNQ4 channel, despite their profound effects on cell surface expression (Fig. 1). Consistent with these findings, previous biochemical studies by others also showed that three of these mutations, W274S, G285C and G296S, had no effect on the cellular level of the KCNQ4 channel 19, 29. Taken together, these data suggest that the DFNA2 mutations L274H, W276S, L281S, G285C, G285S, G296S and G321S do not cause major changes in the KCNQ4 channel structure that are recognizable by ER quality control system 50. Therefore, these mutant channels evade ER-associated degradation, but fail in trafficking to the plasma membrane 47. Intracellular retention, instead of degradation, of the mutant channels provided a molecular basis for the dominant-negative effect of DFNA2 mutations on the WT KCNQ4 function.

Various missense mutations and deletions in the *KCNQ4* gene have been identified in DFNA2 families. A genotype–phenotype correlation has been observed in which missense mutations are associated with a younger onset and severe to profound hearing loss across all frequencies; while deletions are associated with a later onset and milder hearing loss that mainly affects high frequencies 15, 21, 23, 24, 26, 29, 51. To date, our understanding of the molecular basis underlying this genotype–phenotype correlation is limited. Two small deletions identified in DFNA2 patients are frameshift mutations and result in non-functional KCNQ4 subunits that are truncated before the first transmembrane domain and unable to form tetrameric channels with the WT subunits 15, 23. Because half of the KCNQ4 subunits are normal in these heterozygous DFNA2 patients, hearing loss caused by these deletions is most likely because of haploinsufficiency. On the other hand, there is increasing evidence supporting a dominant-negative mechanism for missense mutations and a small inframe deletion c.664_681del 19. Electrophysiological studies have demonstrated that the missense mutations L274H, W276S, L281S, G285C, G285S, G296S and G321S cause loss of KCNQ4 currents with strong dominant-negative effects on the WT KCNQ4 currents, as does the inframe deletion c.664_681del 7, 19, 29, 32. In addition, this and two previous studies have demonstrated that the missense mutations L274H, W276S, L281S, G296S and G321S also lead to a dramatic decrease in KCNQ4 surface expression with strong dominant-negative effects on the WT KCNQ4 subunit 29, 32. Most importantly, our biochemical data showed that none of these DFNA2 mutations affect KCNQ4 subunit interaction, indicating that the mutant KCNQ4 subunits are able to form heteromeric channels with the WT subunit (Fig. 2). As the vast majority of KCNQ4 channels in heterozygous DFNA2 patients would contain at least one mutant subunit (15 of the 16 in theory) 52, it is conceivable that the functional consequence of a missense mutation might be much more profound than that caused by haploinsufficiency. In addition, KCNQ4 channels are expressed in the inner ear and in the central auditory system, where other KCNQ subunits are also present 4, 6, 7, 53. Because KCNQ2, KCNQ3 and KCNQ5 form functional heteromeric channels with KCNQ4 subunits 7, 54, 55, it is likely that a dominant-negative KCNQ4 mutant may also affect the functionality of these KCNQ channels through heteromerization. Collectively, our biochemical data in this study refined our understanding of the molecular mechanism underlying profound hearing loss associated with missense KCNQ4 mutations in the DFNA2 patients.

Sensorineural hearing loss in DFNA2 patients progresses over years 14. The slow progression of DFNA2 implies that KCNQ4 dysfunction in these patients might lead to degenerative processes as observed in many other genetic diseases affecting the nervous system 2. Indeed, studies in mouse models have shown that genetic alterations in *KCNQ4* result in progressive hearing loss that is paralleled by a selective degeneration of the hair cells and spiral ganglion neurons 13. It has been proposed that progressive hearing loss in DFNA2 may result from an increasing load of DFNA2 mutants with age 5. In concordance with this hypothesis, a recent association study in two different human populations has linked *KCNQ4* to age-related hearing loss, which may be attributable to the elevated expression of a causative *KCNQ4* splice variant during ageing 56. The cellular capacity of protein quality control declines during ageing, which is a determining factor in the development and severity of many age-related diseases, such as neurodegenerative diseases, amyotrophic lateral sclerosis, cardiac diseases, cystic fibrosis, type II diabetes, *etc*. 57–59. Thus, it is possible that the increasing load of DFNA2 mutants during ageing may exceed the capacity of the waning protein quality control system; as a result, the mutant KCNQ4 proteins may accumulate excessively and become cytotoxic species. Our findings in this and previous studies support this hypothesis. First, we found that DFNA2 mutants can escape the protein quality control system; they are apparently as stable as the WT channel under normal growth condition (Fig. 1). Second, we have demonstrated that the molecular chaperone HSP90 plays a central role in regulating the cellular level of the KCNQ4 channel 34. HSP90 is well known for its ability in promoting folding and stabilization of mutant proteins and thereby buffers their functional effects. Depletion of HSP90 promotes phenotypic manifestations of genetic and epigenetic variations and has marked effects on the development of various human diseases 35, 59, 60–62. Conceivably, declining protein quality control, especially the decreased cellular level of HSP90, may accelerate the accumulation of cytotoxic DFNA2 mutants in the ageing hair cells and neurons, which in turn lead to cell death and then hearing loss.

Another important but unsolved issue in the molecular aetiology of DFNA2 was whether DFNA2 mutations disrupt the conductance of the KCNQ4 channel, in addition to their effects on KCNQ4 surface expression. Unlike the mutations G285C and G285S, mutations L281S, G296S and G321S do not result in alterations in the signature sequence of the K^+^-filter in the KCNQ4 channel. Thus, it was uncertain whether these intracellularly retained KCNQ4 mutants could assemble channels with normal K^+^ conductance. To answer this question, whole-cell currents were recorded from cells in which the KCNQ4 surface expression had been restored by overexpressing HSP90β. We found that the conductance of the KCNQ4 channel was compromised by these three mutations, as no significant improvement in KCNQ4 current density was observed in these cells. Therefore, our data confirmed that decreased cell surface expression and impaired conductance of the KCNQ4 channel are two independent mechanisms underlying hearing loss in DFNA2; that restoration of KCNQ4 surface expression by overexpression of HSP90 was not sufficient to rescue the channel function in HEK293T cells mimicking the heterozygous condition of DFNA2 patients. These findings together laid important frame work for future studies towards functional rescue of pathogenic KCNQ4 mutations.
